# Association between Maternal Characteristics and Immune Factors TGF-β1, TGF-β2, and IgA in Colostrum: An Exploratory Study in Japan

**DOI:** 10.3390/nu14163255

**Published:** 2022-08-09

**Authors:** Naoko Hirata, Megumi Kiuchi, Kyongsun Pak, Risa Fukuda, Naoko Mochimaru, Mari Mitsui, Kazue Yoshida

**Affiliations:** 1 Department of Product Development, Pigeon Corporation, Ibaraki 300-2495, Japan; 2 Department of Corporate Communications, Pigeon Corporation, Tokyo 103-8480, Japan; 3 Division of Biostatistics, Department of Data Management, Center for Clinical Research and Development, National Center for Child Health and Development, Tokyo 157-8535, Japan; 4 Division of Dermatology, National Center for Child Health and Development, Tokyo 157-8535, Japan; 5 Center for Maternal-Fetal, Neonatal and Reproductive Medicine, National Center for Child Health and Development, Tokyo 157-8535, Japan; 6 Allergy Center, National Center for Child Health and Development, Tokyo 157-8535, Japan

**Keywords:** colostrum, maternal characteristics, immune factors, TGF-β1, TGF-β2, IgA, parity, mode of delivery

## Abstract

Colostrum is the first food for newborns and it contains various crucial immune factors. The concentrations of immune factors in breast milk may change depending on maternal characteristics such as body mass index, collection day, and age at first pregnancy. In this exploratory study, we investigated the association between TGF-β1, TGF-β2, and IgA in colostrum and rarely studied factors that affect breast milk components, including the use of labor-inducing medication, colostrum secretion, sex of newborns, breast or nipple problems, and nipple care. Breast milk samples were collected from 42 mothers and analyzed for TGF-β1, TGF-β2, and IgA. The results suggest that parity and mode of delivery may be correlated with the concentrations of immune factors in colostrum. However, we found no association between the immune factors in colostrum and the use of labor-inducing medications, colostrum secretion, sex of newborns, breast or nipple problems, and nipple care. These findings have some implications for further analysis of the effects of immune factors in breast milk on the prognosis of allergies in children.

## 1. Introduction

Breast milk is often the first source of nutrition for infants, providing irreplaceable nutrients. The World Health Organization (WHO) states that starting breastfeeding within the first postpartum hour is critical to save the life of newborns, and that colostrum is called the baby’s ‘first vaccine’ because it contains antibodies as well as essential nutrients [1].

Immune factors are crucial components of breast milk that cannot be added to artificial milk. Many studies have shown that breast milk contains multiple factors that modulate and promote the development of the infant immune system, including its potential protective role against allergic disease [2] Immune factors in breast milk also supplement the immature immune system of preterm infant, and help prevent inflammatory diseases such as necrotizing enterocolitis (NEC) [3].

There are many types of immune factors in breast milk, and one of them, transforming growth factor-β (TGF-β), is a cytokine that regulates the function of immune cells. TGF-β has been reported to prevent the development of atopic dermatitis in children who are exclusive breastfed [4] and may promote serum IgA production in children [4,5]. TGF-β in breast milk maintains homeostasis in the intestine, regulates inflammation and allergies development in infants, and is important in the development of oral tolerance [6]. TGF-β in breast milk increases when mothers ingest probiotics during pregnancy or lactation [7,8,9]. IgA in breast milk also plays an important role in the maturation of the intestinal immune system of infants and is positively correlated with two isoforms of TGF-β (TGF-β1 and TGF-β2) in colostrum [10,11].

The concentrations of these immune factors may change depending on the maternal characteristics. TGF-β in breast milk is associated with maternal body mass index (BMI) [12], collection day, and age at first pregnancy [13]; IgA in breast milk varies with the time of collection postpartum [14] and place of residence [13]. In addition, there is discussion regarding the differences in TGF-β levels in breast milk depending on the mode of delivery; while TGF-β2 level has been reported to be higher in the breast milk of mothers who deliver via caesarean section than those who deliver vaginally [15], another report suggests that the TGF-β2 level is not related to the mode of delivery [16]. The association between the history of maternal allergic disease and immune factors in colostrum or mature milk is also controversial [10,17,18,19]. According to a systematic review [20], many studies have reported on the relationship between the mother’s diet, supplementary intake, and BMI and breast milk components, especially macronutrients such as lipids, but there remains a lack of information on parity, mode of delivery, age, and medical history. Although various maternal characteristics are related to the immune factors in breast milk, the reported results are inconsistent and warrant further investigation.

In this study, we investigated the association between TGF-β1, TGF-β2, and IgA levels in colostrum and the use of labor-inducing medication, colostrum secretion (secretion before or after birth), sex of newborns, breast or nipple problems, and nipple care, which are rarely studied factors that affect breast milk components, in addition to the previously reported maternal factors associated with immune factors in breast milk.

## 2. Materials and Methods

### 2.1. Subjects

This study involved 42 breastfeeding women aged between 26 to 46 years old who gave birth at the National Center for Child Health and Development between July 2019 and October 2020. Breast milk (colostrum) was collected from all 42 participants. Of these 42 samples, the levels of TGF-β1, TGF-β2, and IgA were measured in 40 samples, while the levels of either TGF-β (TGF-β1 and TGF-β2) or IgA were measured in the remaining 2 samples. Therefore, each factor measured in colostrum had 41 data points. Mature milk was also collected from the same subjects (37 of 42 women, 5 of them dropped out). Similar to the data collected for colostrum, each factor measured in mature milk had 36 data points. Written informed consent for publication of their details was obtained from the study participants.

### 2.2. Sample Collection

The mothers answered questionnaires requesting background information on themselves including their height and body weight before pregnancy, age at delivery, age at first delivery, parity, smoking experience, mode of delivery (vaginal birth or caesarean section), use/non-use of labor-inducing medication, colostrum secretion, sex of newborns, gestational age, history of maternal allergies, breast or nipple problems, and nipple care. Mothers were considered to have a history of allergies if they had been affected by atopic dermatitis, asthma, allergic rhinitis, allergic conjunctivitis, hay fever, or food allergies. Breast or nipple problems were determined based on the presence of swelling, lumps, fever, redness, or scratches on the breast or nipples at the time of the survey. Mothers who had applied a topical agent (oil or cream) to the breasts or nipples at the time of the survey were considered to perform nipple care.

The breast milk sampled between postpartum days 2 and 6 was defined as colostrum, and that sampled between postpartum days 25 and 37 was defined as mature milk in this study. The samples (5 mL) were collected in the test tubes by hand expression with sterilized gloves. The samples were dispensed into 0.5 to 1.0 mL amounts for each factor and then frozen at −80 °C. 

### 2.3. Defatting Treatment of Milk Samples

The frozen breast milk was thawed at room temperature (20–25 °C), and TGF-β1 and TGF-β2 samples were centrifuged at 4 °C and 10,000× *g* for 10 min (Sorvall ST 8R, Thermo Fisher Scientific, Waltham, MA, USA). Of the three separated layers, the intermediate layer was extracted using a 1 mL syringe (SS-01P2725, TERUMO, Tokyo, Japan) equipped with an injection needle at 600 μL per 1 mL of breast milk.

The IgA sample was thawed similarly and centrifuged at 4 °C and 500× *g* for 15 min; 600 μL per 1 mL of breast milk was extracted from the intermediate layer and centrifuged again (4 °C, 3000× *g*, 15 min) before extracting 500 μL of the intermediate layer.

### 2.4. Measurement of TGF-β1 and TGF-β2

To measure TGF-β1 and TGF-β2, the Quantikine human TGF-β1 enzyme linked immunosorbent assay (ELISA) kit (DB100B) and Quantikine human TGF-β2 ELISA Kit (DB250) (both R&D Systems, Inc., Minneapolis, MN, USA) were used, respectively. TGF-β was activated prior to ELISA. For TGF-β1, 80 μL of 1 N HCl was mixed with 160 μL of the previously described defatted milk and incubated at room temperature for 10 min. After the reaction, 80 μL of 1.2 N NaOH/0.5 M HEPES was added to neutralize the solution. The sample was diluted to three ratios of 1:1, 1:2, and 1:4 using the supplied diluent. Subsequent operations were performed according to the procedure manual. A 450 nm wavelength plate reader (Multiskan FC, Thermo Fisher Scientific, Waltham, MA, USA) was used to measure absorbance, and a sample with a standard concentration within the range of 31.3–1000 pg/mL and a low dilution ratio was used. The value obtained by doubling the concentration was taken as the final value of TGF-β1.

For TGF-β2, 25 μL of 1 N HCl was mixed with 125 μL of defatted milk and incubated at room temperature for 10 min. Next, 25 μL of 1.2 N NaOH/0.5 M HEPES was added. This sample was diluted to three ratios of 1:1, 1:2, and 1:4 with the supplied diluent. Following operations were performed according to the procedure manual. The same 450 nm wavelength plate reader was used to take absorbance measurements, and a sample with a standard concentration within the range of 31.3–1000 pg/mL and a low dilution ratio was used. The value obtained by multiplying the concentration by 7.8 was taken as the final value of TGF-β2.

### 2.5. Measurement of IgA

The ab196263-IgA Human ELISA Kit (Abcam) was used for IgA measurement. The defatted milk was diluted to ratios of 1:160,000, 1:320,000, and 1:640,000 using the accompanying diluent to obtain the samples, and subsequent manipulations were performed according to the procedure manual. The same plate reader was used to measure absorbance, and a sample with a standard concentration within the range of 0.78–25 ng/mL and a low dilution ratio was used. The final concentration was shown in terms of mg/mL.

### 2.6. Statistical Analysis

For the immune factors in the colostrum, the median and interquartile range (IQR: 25th–75th percentile points) were calculated for each category of maternal characteristics. To evaluate the differences in the distribution of colostrum components between the categories of maternal characteristics, the Wilcoxon rank-sum test was performed when the characteristics were in two categories, and the Kruskal–Wallis test was performed when they were in three categories.

To further investigate the effect of maternal characteristics on immune factors in the colostrum, a multivariable analysis was performed by applying the forward-backward stepwise method based on the Akaike information criterion (AIC). Each colostrum factor was logarithmically transformed, and a linear regression model was applied. Estimated values of the regression coefficients and geometric mean (regression coefficients converted by inverse logarithm) of the selected factors, their 95% confidence intervals (95% CI), and *p*-values were calculated. All statistical analyses were performed using R version 4.1.2.

## 3. Results

### 3.1. Relationship between Maternal Characteristics and Immune Factors in Colostrum Breast Milk 

Table 1 shows the relationship between the maternal characteristics and the levels of TGF-β1, TGF-β2, and IgA in colostrum. 

Regarding parity, median TGF-β1 levels were 1214.5 pg/mL (IQR: 809.1 to 1873.6) for primipara and 710.3 pg/mL (IQR: 515.4 to 830.1) for multipara; median TGF-β2 levels were 5673.0 pg/mL (IQR: 4093.2 to 12,422.9) for primipara and 4071.0 pg/mL (IQR: 2469.1 to 5227.0) for multipara; thus, TGF-β1 and TGF-β2 levels were higher in primipara than in multipara (*p* = 0.002 and *p* = 0.033, respectively). With respect to the age at delivery, median TGF-β1 levels were 1712.1 pg/mL (IQR: 1030.3 to 2387.9) for women in their 20s, 830.2 pg/mL (IQR: 707.2 to 1175.6) for those in their 30s and 1652.8 pg/mL (IQR: 967.5 to 2167.6) for those in their 40s, and there were significant differences between these age groups (*p* = 0.018). In terms of the age at first delivery, median TGF-β1 levels were 1353.3 pg/mL (IQR: 901.0 to 2131.2) for women who delivered in their 20s, 829.9 pg/mL (IQR: 688.3 to 1027.1) in their 30s and 1652.8 pg/mL (IQR: 967.5 to 2167.6) in their 40s. There were significant differences in TGF-β1 levels between the first delivery age groups (*p* = 0.015). With regard to the mode of delivery, median TGF-β1 levels were 871.9 pg/mL (IQR: 730.7 to 1425.5) for vaginal delivery and 1593.0 pg/mL (IQR: 877.7 to 2272.7) for caesarean section; thus, TGF-β1 level was higher for caesarean section than for vaginal delivery (*p* = 0.102). There were no significant differences in IgA levels between any of the maternal characteristics.

### 3.2. Trends in Colostrum and Mature Milk

**Figure 1:**
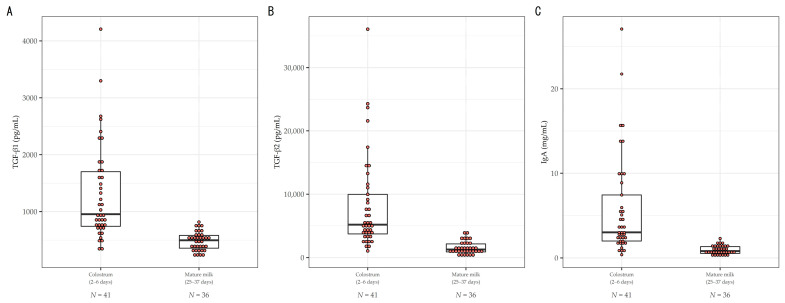
Levels of (**A**) TGF-β1; (**B**) TGF-β2; and (**C**) IgA in colostrum and mature milk. Box plot represent medians with 25th and 75th percentile values, min–max range, and outliers.

Figure 1 shows the distribution of TGF-β1, TGF-β2, and IgA in colostrum (postpartum days 2 to 6) and mature milk (postpartum days 25 to 37) for all 42 subjects. In colostrum, the median TGF-β1 level was 954.7 pg/mL (IQR: 740.1 to 1700.6), median TGF-β2 level was 5173.2 pg/mL (IQR: 3732.9 to 9969.9), and median IgA level was 3.02 mg/mL (IQR: 2.00 to 7.44), whereas in mature milk, the median TGF-β1 level was 498 pg/mL (IQR: 356.6 to 581.4), median TGF-β2 level was 1263.7 pg/mL (IQR: 889.3 to 2141.8), and median IgA level was 0.80 mg/mL (IQR: 0.53 to 1.31). While the variability among subjects was large for all three factors in colostrum, it was smaller in mature milk.

### 3.3. Degree of Influence of Maternal Characteristics

Since the colostrum data in Figure 1 demonstrate large dispersion, we hypothesized that maternal factors before delivery and at the time of delivery may affect the heterogeneity of immune factors in colostrum. Therefore, we studied the maternal factors that would affect the immune factors in colostrum. Table 2 shows the results of multivariable analysis after selecting the variables for each colostrum component using the stepwise method. Regarding the TGF-β1 in colostrum, parity (multipara [reference group: primipara]) with a geometric mean of 0.58 (95%CI: 0.40 to 0.85), mode of delivery (caesarean section [reference group: vaginal delivery]) with a geometric mean of 1.49 (95%CI: 1.08 to 2.05), age at first delivery (30s and 40s [reference group: 20s]) with a geometric means of 0.63 (95% CI: 0.41 to 0.98) and 0.89 (95%CI: 0.56 to 1.44) were chosen, and each variable showed a considerable correlation with the TGF-β1 level (AIC = −5.5, RMSE = 3.99, adjusted R-squared = 0.356). Regarding TGF-β2 in colostrum, parity (multipara [reference group: primipara]) with a geometric mean of 0.40 (95%CI: 0.24 to 0.68), mode of delivery (caesarean section [reference group: vaginal delivery]) with a geometric mean of 1.74 (95%CI: 1.08 to 2.79), collection day (4 d or ≥5 d [reference group: ≤3 d]) with geometric mean of 0.65 (95%CI: 0.35 to 1.19) and 0.62 (95%CI: 0.37 to 1.02), and history of maternal allergy (yes [reference group: no]) with a geometric mean of 0.63 (95%CI: 0.38 to 1.06) were selected, and parity and mode of delivery were significantly correlated with the TGF-β2 level (AIC = 24.4, RMSE = 4.96, adjusted R-squared = 0.250). Regarding IgA in colostrum, collection day (4 d or ≥5 d [reference group: <3 d old]) with geometric mean of 0.67 (95%CI: 0.30 to 1.49) and 0.37 (95%CI: 0.19 to 0.72), mode of delivery (caesarean section [reference group: vaginal delivery]) with a geometric mean of 2.03 (95%CI: 1.06 to 3.90), pre-pregnancy BMI (≥18.5, <25.0 kg/m^2^ and ≥25.0 kg/m^2^ [reference group: <18.5 kg/m^2^]) with a geometric mean of 0.51 (95%CI: 0.20 to 1.33) and 0.25 (95%CI: 0.06 to 1.02), and use of labor-inducing medication (yes [reference group: no]) with a geometric mean of 1.87 (95%CI: 1.00 to 3.50) were selected. The mode of delivery and collection day were considerably correlated with the IgA level (AIC = 46.6, RMSE = 1.14, adjusted R-squared = 0.140).

## 4. Discussion

The results of this study suggest that parity and mode of delivery may be correlated with the concentrations of TGF-β1, TGF-β2, and IgA in colostrum. In contrast, the use/non-use of labor-inducing medication, colostrum secretion, sex of newborns, breast or nipple problems, and nipple care were not related to TGF-β1, TGF-β2, and IgA levels in colostrum.

The TGF-β1 and TGF-β2 concentrations in colostrum tended to be higher in pri-mipara than in multipara (Table 2). Primipara tend to produce less breast milk than multipara during the early postpartum period [21]. Assuming that the amount of TGF-β produced is similar in primipara and multipara, it is possible that TGF-β1 and TGF-β2 in the colostrum of primipara are concentrated and higher in volume than those in multipara. This result is consistent with a previous report [22]. TGF-β is also involved in mammary gland regression [23,24], which may have affected the TGF-β concentration in colostrum in multipara who have had experience of this regression.

The mode of delivery affected the concentrations of TGF-β1, TGF-β2, and IgA in the colostrum; the concentrations were higher for subjects who delivered by caesarean section than for those who delivered vaginally (Table 2). During skin wound healing, both TGF-β1 and TGF-β2 act on inflammation, angiogenesis, re-epithelialization, and connective tissue regeneration [25]. Therefore, TGF-β1 and TGF-β2 levels may increase in the blood and may be secreted into the breast milk during the process of wound healing after caesarean section. However, the relationship between TGF-β concentration in breast milk and caesarean section is still being debated, with some reports showing no significant difference in TGF-β concentrations in colostrum after caesarean section [12,13,16], and others indicating no correlation between TGF-β concentrations in breast milk and plasma [26]. It is possible that the concentrations become temporarily high during tissue repair, but our results were consistent with the relationship between mode of delivery and TGF-β2 in breast milk reported by Kociszewska–Najman et al. [15].

With regard to the collection day, the IgA concentration in the colostrum was lower on day 5 than on day 3 (Table 2), which was consistent with the decrease in the IgA concentration over time postpartum [14]. Furthermore, the concentrations were lower when the age at first delivery was in the 30s than when it was in the 20s (Table 2). The TGF-β1 concentration in breast milk is likely to decrease, which might be due to protein concentrations in breast milk decreasing with age [27]. Changes in mammary epithelial structure and galactopoiesis with age [28] may also have an impact on TGF-β1 concentrations. The concentrations are higher when the age at first delivery is in the 40s than when it is in the 30s. Since there was no multipara among the subjects in the 40s, this may have been caused by the parity rather than the age itself. In Japan, the age at first delivery has been increasing in recent years; thus, such situations are not rare [29].

Breast milk concentrations may be affected by diurnal variations [30,31], inter-day variations, and breast fullness [32], in addition to the maternal factors at childbirth and before childbirth. Since we did not want to burden the subjects after delivery, we could not sample breast milk in a manner that would not be affected by diurnal and inter-day variations. Although it is difficult to control the conditions for breast milk collection, it is necessary to acquire additional data on diurnal and inter-day fluctuations regarding the factors of parity and mode of delivery. This was an exploratory study in which the sample size was defined by the study feasibility; consequently, the statistical power may be insufficient.

Our results suggest that TGF-β concentrations in colostrum tend to be higher in primipara and caesarean section cases. Primipara who deliver by caesarean section may experience extra physical burden during delivery, which may delay the start of lactation [21,33]. Therefore, appropriate lactation guidance and support with a breast pump may help newborns to intake immune factors in colostrum. 

We have reported on the association between TGF-β and IgA concentrations in colostrum and maternal characteristics, and we will continue to measure the immune factors in breast milk after childbirth and collect data on allergies and skin conditions in infants until one year old. Furthermore, we will study the effects of immune factors in breast milk on the prognosis of allergies in children.

## 5. Conclusions

Our investigation suggests that parity and mode of delivery may be correlated with the concentrations of TGF-β1, TGF-β2, and IgA in colostrum. However, we found no association between the TGF-β1, TGF-β2, and IgA levels in colostrum and the use/non-use of labor-inducing medications, colostrum secretion, sex of newborns, breast or nipple problems and nipple care.

## Figures and Tables

**Table 1:** Maternal characteristics and levels of TGF-β1,2 and IgA in the colostrum.

Factors	Classes	N (%)	TGF-β1 (pg/mL)		TGF-β2 (pg/mL)		IgA (mg/mL)	
			Median (IQR)	*p*-Value	Median (IQR)	*p*-Value	Median (IQR)	*p*-Value
BMI before pregnancy	<18.5 kg/m^2^	4 (9.5%)	726.4 (601.4 to 1446.9)	0.781	2474.4 (2433.4 to 4759.7)	0.258	4.34 (3.11 to 10.57)	0.664
	≥18.5, <25.0 kg/m^2^	35 (83.3%)	1070.3 (774.5 to 1676.7)	-	5247.5 (4073.8 to 9768.3)	-	3.30 (2.04 to 8.51)	-
	≥25.0 kg/m^2^	3 (7.1%)	954.7 (657.5 to 2580.4)	-	6843.9 (5048.1 to 21,453.3)	-	2.86 (2.43 to 2.91)	-
Age at delivery	20s	4 (9.5%)	1712.1 (1030.3 to 2387.9)	0.018 *	5070.8 (4284.6 to 6545.6)	0.320	4.55 (4.29 to 5.92)	0.340
	30s	24 (57.1%)	830.2 (707.2 to 1175.6)	-	4889.7 (3269.6 to 7606.2)	-	2.55 (1.86 to 5.45)	-
	40s	14 (33.3%)	1652.8 (967.5 to 2167.6)	-	8721.2 (4120.8 to 14,214.8)	-	3.61 (2.86 to 9.85)	-
Age at first delivery	20s	6 (14.3%)	1353.3 (901.0 to 2131.2)	0.015 *	5070.8 (3916.8 to 7902.5)	0.330	4.55 (3.81 to 5.30)	0.499
	30s	22 (52.4%)	829.9 (688.3 to 1027.1)	-	4889.7 (3286.8 to 7541.2)	-	2.55 (1.89 to 5.33)	-
	40s	14 (33.3%)	1652.8 (967.5 to 2167.6)	-	8721.2 (4120.8 to 14,214.8)	-	3.61 (2.86 to 9.85)	-
Smoking experience	No	33 (78.6%)	915.7 (730.7 to 1511.4)	0.164	5057.8 (3643.1 to 8775.1)	0.485	3.30 (2.25 to 6.38)	0.889
	Yes	9 (21.4%)	1700.6 (830.2 to 2272.7)	-	6418.5 (4054.4 to 13,261.9)	-	2.86 (1.95 to 7.44)	-
Mode of delivery	Vaginal delivery	28 (66.7%)	871.9 (730.7 to 1425.5)	0.102	4643.0 (3278.2 to 7946.8)	0.096	3.02 (1.86 to 6.69)	0.596
	Caesarean section	14 (33.3%)	1593.0 (877.7 to 2272.7)	-	7671.2 (4889.7 to 14,532.4)	-	3.24 (2.50 to 8.04)	-
Labor-inducing medication	Non-use	21 (50.0%)	898.2 (721.3 to 1511.4)	0.358	5057.8 (3151.5 to 8976.7)	0.579	2.68 (1.81 to 6.38)	0.282
	Use	21 (50.0%)	1027.1 (772.5 to 1852.3)	-	5673.0 (4054.4 to 11,023.8)	-	3.61 (2.65 to 7.44)	-
Colostrum secretion	Before birth	8 (19.0%)	846.6 (689.4 to 1060.0)	0.306	3880.7 (3081.0 to 4555.7)	0.058	2.67 (2.21 to 3.95)	0.339
	After birth	34 (81.0%)	1113.6 (772.5 to 1740.1)	-	5673.0 (4132.1 to 11,023.8)	-	3.61 (2.00 to 8.87)	-
Collection day	≤3 d	15 (35.7%)	1132.2 (710.3 to 1544.6)	0.830	5437.1 (4537.3 to 11,212.7)	0.773	5.07 (2.62 to 9.94)	0.242
	4 d	9 (21.4%)	954.7 (787.0 to 2272.7)	-	4397.6 (3732.9 to 6843.9)	-	3.27 (1.98 to 10.95)	-
	≥5 d	18 (42.9%)	877.7 (740.1 to 1852.3)	-	4889.7 (3286.8 to 9969.9)	-	2.75 (1.80 to 4.35)	-
Parity	Primipara	32 (76.2%)	1214.5 (809.1 to 1873.6)	0.002 **	5673.0 (4093.2 to 12,422.9)	0.033 *	3.02 (2.16 to 6.69)	0.823
	Multipara	10 (23.8%)	710.3 (515.4 to 830.1)	-	4071.0 (2469.1 to 5227.0)	-	4.01 (2.01 to 8.04)	-
Sex of newborns	Male	22 (52.4%)	915.7 (697.7 to 1525.8)	0.248	4643.0 (2850.2 to 8369.5)	0.293	2.95 (2.00 to 5.93)	0.633
	Female	20 (47.6%)	1132.2 (808.5 to 1986.7)	-	5673.0 (4264.8 to 10,776.9)	-	3.64 (2.24 to 7.80)	-
History of maternal allergies	Yes	33 (78.6%)	990.9 (721.3 to 1710.5)	0.722	4995.3 (3352.0 to 8775.1)	0.154	3.59 (1.98 to 8.04)	0.676
	No	9 (21.4%)	877.7 (829.9 to 1605.0)	-	7671.2 (4889.7 to 14,532.4)	-	2.90 (2.33 to 5.41)	-
Breast or nipple problems	Yes	30 (71.4%)	1027.1 (772.5 to 1740.1)	0.661	5321.8 (3373.7 to 11,023.8)	0.966	2.95 (1.95 to 5.55)	0.205
	No	12 (28.6%)	853.8 (730.7 to 1394.3)	-	5057.8 (4350.3 to 6140.1)	-	4.82 (2.89 to 10.80)	-
Nipple care	Yes	23 (54.8%)	1310.3 (762.9 to 1730.2)	0.237	6045.8 (3537.4 to 12,842.4)	0.460	3.65 (2.55 to 5.84)	0.331
	No	19 (45.2%)	830.2 (752.4 to 1175.6)	-	4889.7 (3893.6 to 7192.6)	-	2.65 (1.76 to 9.42)	-

There are significant differences between each class (* *p* < 0.05; ** *p* < 0.01); The gestational age of the 42 subjects were such that 1 subject was <37 weeks (2.4%) and 41 subjects were 38 weeks or more (97.6%), and almost all subjects had full-term birth. The statistical analysis involved 41 subjects because of the insufficient volume of breast milk and missing values depending on maternal factors. With regards to age, 20s indicates ages 20 to 29, 30s indicates ages 30 to 39, while 40s indicate ages 40 and above.

**Table 2:** Variables selected via forward-backward stepwise selection and the coefficients of multivariable linear regression model for log-transformed data.

	Variable	Coefficients (95%CI)	Geometric Mean (95%CI)	*p*-Value
TGF-β1	Intercept	3.40 (3.12 to 3.67)	2494.6 (1325.8 to 4693.7)	<0.001
	Age at first delivery (Ref: 20s): 30s	−0.20 (−0.39 to −0.01)	0.63 (0.41 to 0.98)	0.046 *
	:40s	−0.05 (−0.26 to 0.16)	0.89 (0.56 to 1.44)	0.650
	Mode of delivery (Ref: Vaginal birth): Caesarean section	0.17 (0.03 to 0.31)	1.49 (1.08 to 2.05)	0.020 *
	Parity (Ref: Primipara): Multipara	−0.24 (−0.40 to −0.07)	0.58 (0.40 to 0.85)	0.008 **
TGF-β2	Intercept	4.47 (4.07 to 4.88)	29,789.1 (11,637.9 to 76,250.2)	<0.001
	Mode of delivery (Ref: Vaginal delivery): Caesarean section	0.24 (0.03 to 0.45)	1.74 (1.08 to 2.79)	0.029 *
	Collection day (Ref: ≤3 d): 4 d	−0.19 (−0.45 to 0.07)	0.65 (0.35 to 1.19)	0.169
	:≥5 d	−0.21 (−0.43 to 0.01)	0.62 (0.37 to 1.02)	0.071
	Parity (Ref: Primipara): Multipara	−0.40 (−0.63 to −0.17)	0.40 (0.24 to 0.68)	0.002 **
	History of maternal allergies (Ref: No): Yes	−0.20 (−0.42 to 0.03)	0.63 (0.38 to 1.06)	0.093
IgA	Intercept	0.84 (0.39 to 1.28)	6.85 (2.46 to 19.03)	<0.001
	BMI before pregnancy (Ref: <18.5 kg/m^2^): ≥18.5, <25.0 kg/m^2^	−0.29 (−0.70 to 0.12)	0.51 (0.20 to 1.33)	0.179
	:≥25.0 kg/m^2^	−0.60 (−1.21 to 0.01)	0.25 (0.06 to 1.02)	0.062
	Mode of delivery (Ref: Vaginal delivery): Caesarean section	0.31 (0.02 to 0.59)	2.03 (1.06 to 3.90)	0.041 *
	Labor-inducing medication (Ref: Without): With	0.27 (0.00 to 0.54)	1.87 (1.00 to 3.50)	0.059
	Collection day (Ref: ≤3 d): 4 d	−0.17 (−0.52 to 0.17)	0.67 (0.30 to 1.49)	0.334
	:≥5 d	−0.43 (−0.72 to −0.14)	0.37 (0.19 to 0.72)	0.006 **

The estimated coefficients in log10 scale and the geometric mean and their corresponding 95% confidence intervals are presented. * *p* < 0.05, ** *p* < 0.01; The statistical analysis involved 41 subjects because of the insufficient volume of breast milk and missing values depending on maternal factors.

## Data Availability

Data will be provided upon request.
